# Molecular characterization of human calicivirus associated with acute diarrheal disease in mexican children

**DOI:** 10.1186/1743-422X-9-54

**Published:** 2012-02-23

**Authors:** Fabián Gómez-Santiago, Rosa María Ribas-Aparicio, Herlinda García-Lozano

**Affiliations:** 1 Laboratorio de Virus Gastrointestinales, Departamento de Virología del Instituto de Diagnóstico y Referencia Epidemiológicos (InDRE), Secretaría de Salud (SSa), Carpio 470, Col. Santo Tomás, 11340 México, DF, Mexico; 2 Laboratorio de Producción y Control de Biológicos. Departamento de Microbiología, Escuela Nacional de Ciencias Biológicas (ENCB), Instituto Politécnico Nacional (IPN), Mexico City, Mexico

**Keywords:** Norovirus, Sapovirus, *RdRp *gene, Capsid gene, Molecular genotyping, Gastroenteritis, Phylogeny

## Abstract

**Background:**

Human caliciviruses (HuCV) are emerging enteric pathogens that are a common cause of diarrhea in humans worldwide. Due to the paucity of information on the molecular characterization of HuCV circulating in Mexico, the aim of this work was to investigate the diversity and molecular epidemiology of the HuCV infection associated with acute diarrheal disease in Mexican children aged up to 5 years.

**Results:**

Of the 131/414 (32%) HuCV positive-specimens analyzed, 128 were identified as Norovirus (NoV) and three as Sapovirus (SaV). Of the NoV positive specimens, 118/128 (92%) were NoV GII and 10/128(8%) were untypeable by RT-PCR in both polymerase and capsid genes, whereas one SaV isolate was further confirmed by sequencing as GI.2. Phylogenetic analysis based on polymerase partial gene sequences from 89/131 (68%) HuCV isolates showed that 86/89 (97%) belong to NoV GII.4 with three main variant clusters of this genotype, 2/89 (2%) to NoV GII.2, and 1/89 (1%) to SaV GI.2. Furthermore, partial sequencing of the capsid gene *VP1 *of 63/131 (48%) strains indicated that 61/63 (97%) correlated with NoV GII.4, whereas only 2/63 (3%) clustered to NoV GII.2. HuCV infections were detected throughout the year, and the highest number of cases positive for NoV was found in children between 7 and 18 months of age (60%).

**Conclusions:**

This study highlights the usefulness of analyzing both polymerase and capsid genes for molecular characterization of HuCV and demonstrates the relatedness and predominance of NoV GII.4 with acute diarrheal disease in young Mexican children, thus contributing to better understanding of the molecular epidemiology of this disease.

## Background

Acute gastroenteritis remains a major public health problem worldwide. Recently, mortality due to diarrheal disease has been reported in > 1 million human deaths annually, with young children comprising the most important age group affected [1]. Human caliciviruses (HuCV) include the genera Norovirus (NoV) and Sapovirus (SaV); in particular, NoV has been recognized as the most important cause of nonbacterial, acute gastroenteritis in humans of all age groups. Additionally, NoV is responsible for at least 50% of all gastroenteritis outbreaks globally; however, the incidence of this agent is rarely laboratory-registered in developing countries [2].

NoV are genetically diverse; 35 different genotypes are now classified within five genogroups (GI-GV) based on their capsid and/or polymerase genes: 14 genetic genotypes in GI; 17 in GII; two in GIII; one in GIV, and one in GV [3,4]. The SaV genus is divided into five genogroups and at least 16 genetic clusters. Genogroups I, II, IV, and V infect humans, whereas genogroup III infects only animals [5]. SaV strains that belong to genogroup I have been detected predominantly over the last decade in Japan, whereas NoV GII.4 is the major cause of acute, nonbacterial gastroenteritis and food-related outbreaks circulating worldwide [5,6].

The highly genetic diversity that HuCV exhibit has made it complicated to develop a universal system for their classification. Reverse transcription-polymerase chain reaction (RT-PCR) assays [7], as well as Enzyme-linked immunosorbent assays (ELISA), have been extensively employed for their detection [8]. Usually, the genomic region utilized to detect and genotype HuCV by RT-PCR codify to the polymerase gene (*RdRp*), which is relatively conserved in both genera [9]. Nevertheless, the capsid gene has been useful in obtaining better phylogenetic and genotyping analysis [10].

Some studies have investigated the seroprevalence [11-13] and the genetic diversity [14] of some HuCV circulating strains in Mexico. Additionally, a few have reported NoV strain diversity in asymptomatic children [15] and as causing traveler's disease [16,17]. Furthermore, a study carried out in three Mexico City hospitals characterized some HuCV strains [18]. However, little is known concerning the molecular characterization of HuCV across the country. Thus, this work provides, to our knowledge for the first time important information on the molecular characterization in both genes of NoV strains of different states throughout Mexico for further understanding of the genetic diversity and molecular epidemiology of HuCV infection associated with acute diarrheal disease in Mexican children up to 5 years of age. Moreover, we also describe the predominance of NoV GII.4 strains in children with acute diarrheal disease.

## Results

### Detection of human calicivirus infections

During the study period, a total of 414 stool specimens from pediatric patients with diarrhea, negative to rotavirus analyzed by PAGE in the National Network of Public Health Laboratories (RNLSP, its acronym in Spanish) as well as negative for bacterial and parasitic enteric pathogens, were analyzed. Rotavirus detection by ELISA and RT-PCR yielded 9% (39/414) positive samples employing these assays (data not shown). NoV (128/414, 31%) and SaV (*n *= 3/414, 1%) were detected by RT-PCR with primers targeted toward the *RdRp *gene. The samples positive for NoV corresponded to a DNA fragment of 319 bp, while strains of SaV gave rise to a DNA fragment of higher molecular size of 331 bp.

**Figure 1:**
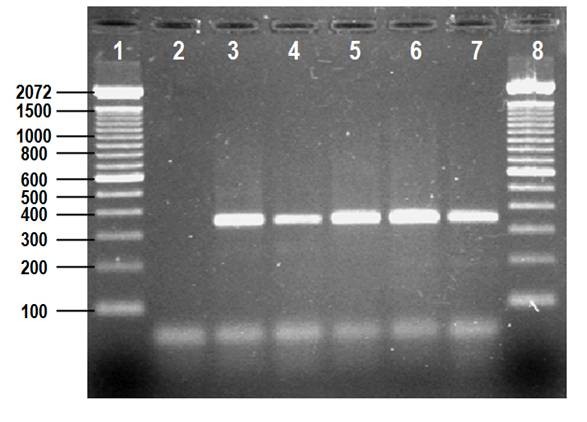
**Genotyping of NoV using the capsid gene**. RT-PCR products of 344 bp correspond to NoV GII (lanes 4, 7). Lane 1 and 8 are DNA molecular size markers. Lanes 2 and 3 are negative and positive controls, respectively.

The RT-PCR reaction performed to genotype the 128 NoV-positive samples employing primers targeted toward the *RdRp *gene indicated that 72/128 (56%) viral strains belong to NoV GII, whereas no sample was amplified for NoV GI or for a combination of GI and GII. However, of 56 NoV positive samples that were negative for the *RdRp *genotyping reaction, it was possible to identify 46/56 samples for a total of 118/128 for NoV GII, with primers targeted toward the capsid gene (Table 1) (Figure 1).

**Table 1:** Identification of HuCV detected by RT-PCR associated with acute diarrhea illness in Mexico

			Genotyping						
		
			Primers *RdRp*		Primers capsid		Primers *RdRp*	Primers *RdRp*/capsid	
**Period of collection of specimens**	**Total samples**	**Samples positive for HuCV**	**NoV GI**	**NoV GII**	**NoV GI**	**NoV GII**	**SaV GI**	**NoV GII-GI**	**Negatives to genotyping reaction**

October 2005-December 2006.	414	131	0	72	0	46	3	0	10

### Phylogenetic analysis

**Figure 2:**
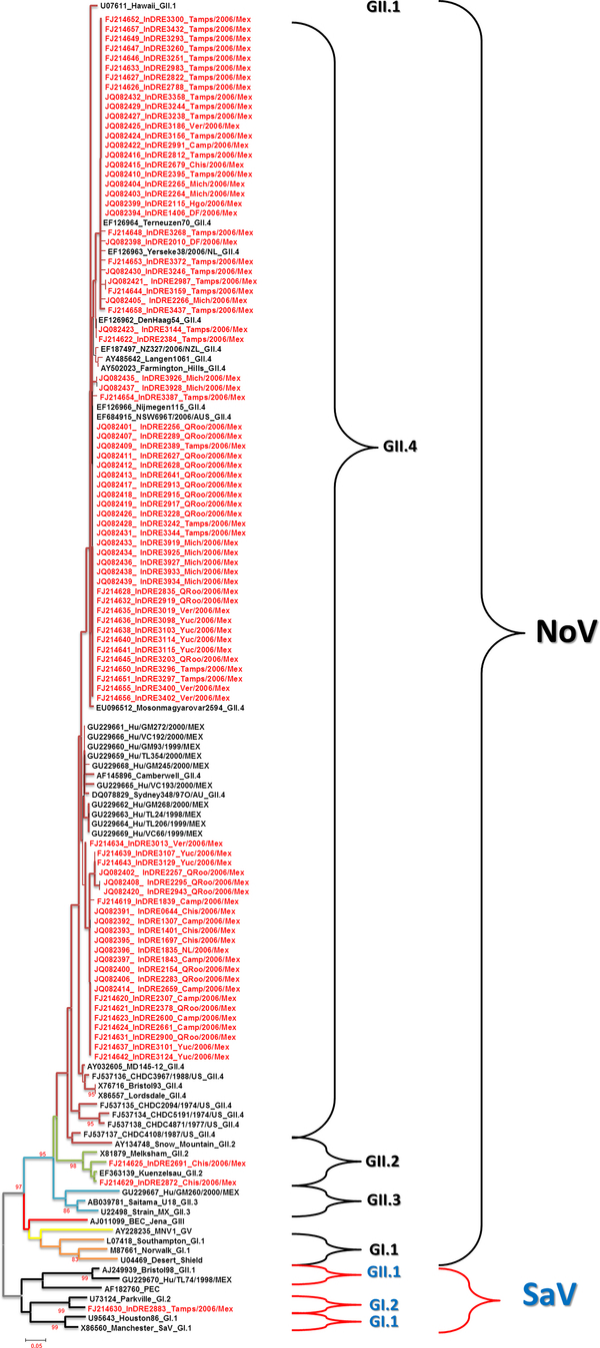
**Phylogenetic tree based on partial *RdRp *gene sequence from 89 human calicivirus (HuCV) isolates identified in this study**. The numbers on the branches indicate the bootstrap values for clusters, indicated as % of 1,000 replicates using the neighbor-joining method with the p-distance model. The distance scale in nucleotide substitutions per position is shown.

**Figure 3:**
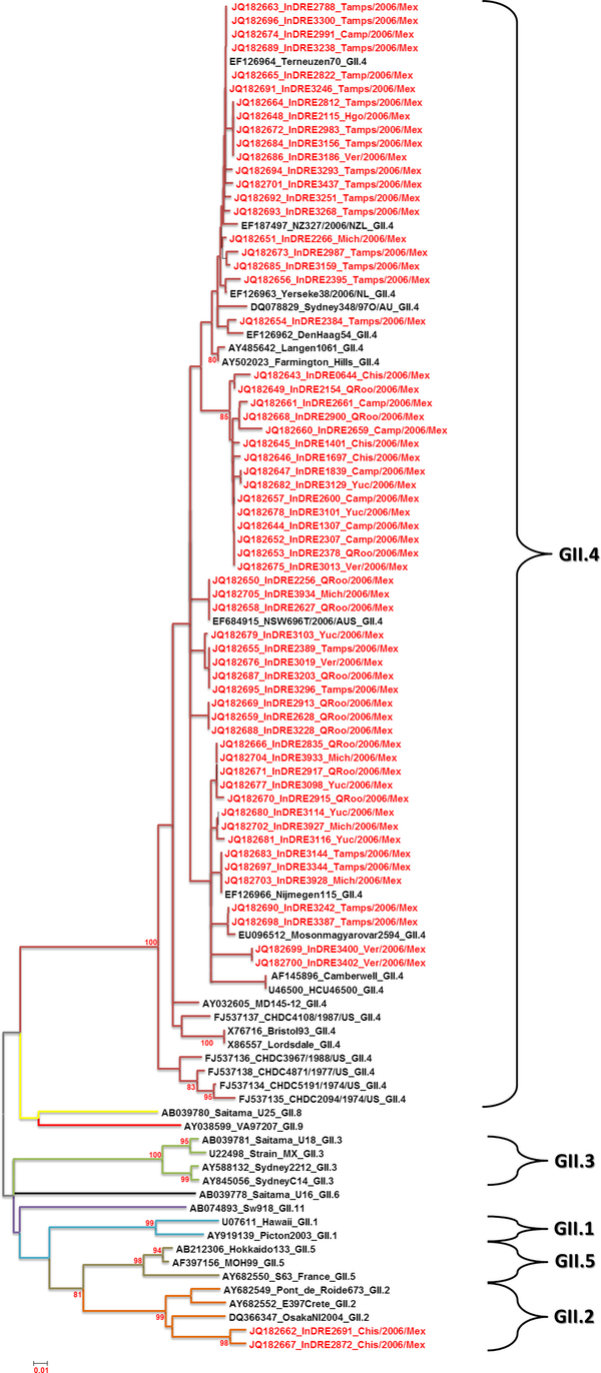
**Phylogenetic tree based on partial *VP1 *capsid gene sequence from 63 Norovirus (NoV) isolates identified in this study**. The numbers on the branches indicate the bootstrap values for clusters, indicated as % of 1,000 replicates using the neighbor-joining method with the p-distance model. The distance scale in nucleotide substitutions per position is shown.

Nucleotide sequence and phylogenetic analysis of 89/131 (68%) HuCV amplicons allowed us to construct a phylogenetic tree based on a 319 bp region of the *RdRp *gene from viral isolates by RT-PCR, revealing 88 NoV GII and one SaV GI. Of the 88 samples corresponding to NoV GII, 86 viral isolates clustered as NoV GII.4 together with another sequences reported at the GenBank, whereas the remaining two showed a close relationship with the Kuenzelsau 3870 and Melksham viral strains, both of which are NoV GII.2. One viral isolate clustered with the SaV viral strain Parkville 94 GI.2 (Figure 2). Interestingly, NoV GII.4 isolates were arranged in three main variant clusters. On the other hand, phylogenetic analysis of 63/131 (48%) amplicons of the *VP1 *capsid partial gene of NoV, indicated that 61 samples belong to genotype NoV GII.4 and that two samples grouped with OsakaNI2004, Point de Roide673 and E397Crete strains reported at the GenBank as NoV GII.2 (Figure 3). *VP1 *phylogenetic tree analysis also revealed several subcluster variants for the GII.4 genotype.

**Figure 4:**
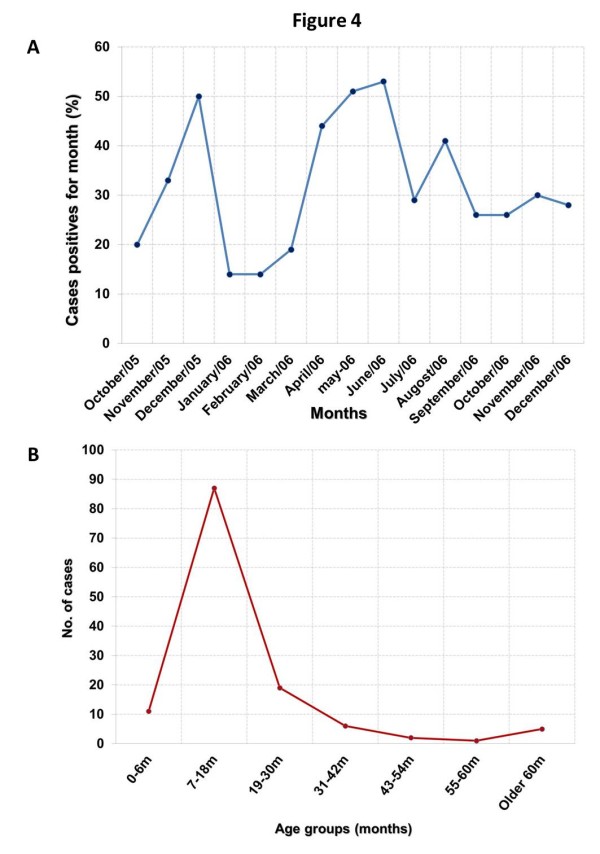
**Seasonality and frequency of human calicivirus (HuCV) infection**. A) Monthly distribution of HuCV-associated with acute gastroenteritis in Mexico from October 2005 to December 2006. B) Frequency of infection of HuCV in Mexican children of different age groups.

NoV infection was detected all year around; however, the number of HuCV detected exhibited two peaks: November-December, and April-June (Figure 4A). Furthermore, the NoV infection was found mainly in children within the age range of 7-8 months (60%) and with low frequency in children from 18 months up to 5 years of age (Figure 4B).

## Discussion

Diversity studies on NoV prevalence and the distribution of genotypes focus on outbreaks [2,3,6]. The aim of this study was to detect HuCV associated with acute diarrheal disease in children using RT-PCR as the principal molecular tool for the detection and genotyping of these viral agents from different Mexico geographic areas that referred cases of acute gastroenteritis in children. Additionally, this assay will be useful at RNLSP to extend national diagnostic coverage of viral gastroenteritis in Mexico.

Using a primer mix [14] targeted toward the RdRp region that contains relatively conserved sequences in both genera, we found HuCV in 32% of the total samples analyzed. These results are in agreement with previous studies carried out in Brazil and Argentina [19,20]. We even utilized a set of primers designed for genotyping the *RdRp *gene in NoV GI and NoV GII, the most common genogroups of NoV; we found samples negative for the first genotyping reaction, and a second set of primers targeted toward the capsid gene was utilized to identify NoV GI and NoV GII specimens. This assay permitted us to increase detection up to a total of 118/128 (92%) of NoV GII. These results indicate the convenience of analyzing *RdRp *and *VP1 *capsid genes routinely to genotype NoV GI and NoV GII strains. Moreover, this could aid in identifying new recombination strains circulating worldwide and to evidence the highly genetic diversity that these viruses exhibit in Mexico. However, we were unable to genogroup 10 samples in both genes, suggesting the presence of other genogroups of NoV in the country. In this study, we tested all 414 samples by ELISA for rotavirus and found 39/414 (9%) positive samples, among which only two samples co-infected with NoV GII 2/414 (0.4%); this rate was much lower than that found in other previously reported studies in Mexico in which the rate of co-infection included 35-75% of samples with other enteric pathogens in international traveler's diarrhea [16,17].

Other studies of HuCV incidence in Mexico have been reported; however, these were carried out in rural communities near Mexico City or in Mexico City hospitals [11,13,18], but limited information existed at the time on molecular epidemiology throughout the country. A few years ago, García et al. found NoV GI and NoV GII in asymptomatic infections in children by utilizing capsid gene amplification [15] without considering cases associated with acute diarrheal disease. Additionally, the frequency of NoV GI as a cause of traveler's diarrhea in international travelers who visited Mexico has been reported as the second most commonly identified enteric pathogen after the enterotoxigenic *Escherichia coli *[16,17]. In this work, 32% of HuCV, specifically NoV GII (31%) and SaV (1%), were found. These data correlate with previous studies that showed a higher proportion of NoV GII than of NoV GI strains in outbreaks and sporadic cases worldwide [21,22]. To our knowledge, this is the first report indicating the frequency of HuCV related with acute diarrheal disease in children up to 5 years of age throughout Mexico and the first time to our knowledge that both genes have been analyzed by sample. Moreover, it proves the need for suitable molecular tools to obtain better understanding of this viral infection to prevent spreading as soon as the disease breaks out.

At present, NoV GII is divided in 17 genotypes, with GII.4 the genotype most commonly associated with many global epidemics of acute gastroenteritis. Outbreaks implicating this genotype have been reported throughout the last decade in several countries all over the world [20,22-25]. Moreover, variants of this genotype were also reported in New Zealand and Australia [26,27]. In accordance with these findings, many reports describe that GII.4 is also the cause of sporadic cases of pediatric gastroenteritis worldwide [21,23,27,28]. These events support the global impact that NoV GII.4 exerts on five continents. The explanation concerning the increased number of NoV outbreaks worldwide could be the rapid evolution of the major capsid protein of GII.4 strains, resulting in new epidemic strains with altered antigenicity. These changes occur mainly on the distal surface of the P2 subdomain, the site involved in virus attachment prior entry into the host cells, and these strains have proven to evolve under the pressure of population immunity [6].

In this study, we detected 32% of HuCV-positives; this could be explained by the ease with which NoV is transmitted by water, food, direct contact, airborne droplets, and vomit, persistence in the environment as a source of contamination, the low infectious dose required to cause illness, and viral resistance to the disinfection process on surfaces, as well as to management and disease prevention in the community [29,30].

During the past 5 years, many strains that clustered with the NoV GII.4 genotype using the sequence of the capsid gene or the *RdRp *gene, including several recombinant strains, have been reported in numerous studies [31-33]. Sequence analysis of short amplicons of the *RdRp *gene showed that the majority of the samples analyzed in this work, 86/89 (97%), belong to NoV GII.4, confirming that the frequency of this genotype in Mexico is in agreement with that of other studies worldwide. In particular, a 63-sequence group, clustered together with Mosonmagyarovar2594, Njimegen115, DenHaag54, Yerseke38, Terneuzen70, and Langen1061 strains, belongs to a cluster of Farmington Hills NoV GII.4 variants, while another 23-sequence group showed high similarity (94-98%) with Sidney348 and 10 additional sequences that have been reported in Mexico City-based hospitals [18], suggesting a close phylogenetic relationship with the Camberwell GII.4 variant that emerged in the 1980s and that likely remained as one of the most predominant groups for several decades [34]. Only two samples collected from Chiapas state exhibited high similarities (90-99%) with the Melksham and Kuenzelsau 3870 GII.2 reference strains, indicating that at least two different NoV genotypes are circulating in Mexico. In this report, SaV was sequenced in one sample that was collected in the state of Tamaulipas, and its phylogenic analysis showed 92% similarity with the viral strain Parkville 94, which belongs to genotype GI.2 of SaV, confirming previous reports that SaV circulates in Mexico [18]. Interestingly, our *RdRp *phylogenetic tree (Figure 2) showed differences in two strains previously reported to be genotyped as NoV GII.4 and SaV GI.2 [18]. Overall, these results suggest a more extensive analysis with larger DNA fragments or the simultaneous analysis of different genomic regions to discern this disparity.

The phylogenetic tree generated with short amplicons of the *VP1 *partial gene indicated that 61 samples clustered with Terneuzen70, NZ327, Yerseke38, Sidney348, DenHaag54, NSW696T, Nijmegen115, and Mosonmagyarovar2594 strains of NoV, which belong to genotype GII.4. Although the percentage of similarity was highly among these (92-100%), several subclusters were found in this genotype, indicating a pronounced genetic variability in the *VP1 *gene. Moreover, two strains designated InDRE2691 and InDRE 2872 clustered with Point de Roide673 and E397Crete strains, which are classified as genotype NoV GII.2, showed a similarity of 89-91%. This phylogenetic analysis confirms the results obtained with the *RdRp *dendrogram, in which these samples also clustered with Melksham and Kuenselzau strains, both belonging to genotype GII.2 of NoV, and demonstrate that the *VP1 *gene of NoV strains GII.4 clusters with pandemic strains such as Sydney348, NZ327/06, and NSW696T/06 [35].

Tamaulipas showed the highest sample number associated with HuCV infection, with 52 (40%) samples positive for NoV and one (1%) for SaV, due to the high frequency of acute gastroenteritis in children in these states during the period of study.

Despite the limited number of positive samples to determine seasonality, distribution of HuCV infection occurred mainly during two periods: November-December, and April-June, in contrast with Brazil, where infection has been reported along the entire year without a seasonal pattern [36]. Interestingly, the age group most affected with HuCV infection (7, 18 months) corresponded to the group most commonly affected by rotavirus, with a median age of 12 months.

Some difficulties in establishing the diagnosis of NoV infection have been the lack of a rapid and sensitive diagnostic method for use in public health laboratories and hospitals; moreover, no specific surveillance system, such as the Rotavirus Surveillance Network, exists in Mexico for HuCV infection or for NoV outbreaks. Consequently, the frequency of HuCV infections is underestimated. However, although the RT-PCR test for diagnosis of these HuCV is the gold standard, it is not routinely performed on all stool specimens negative for rotavirus, despite that NoV are a common cause of infectious gastroenteritis.

Due to that acute diarrhea remains a major public health problem in Latin America, detection of the NoV infection could improve the diagnostic coverage of viral gastroenteritis in children < 5 years of age, because this is the major age group affected by this disease [1].

## Conclusions

This study highlights the usefulness of analyzing both the *RdRp *and capsid genes for molecular characterization of HuCV and demonstrates the relatedness and predominance of NoV GII.4 with acute diarrheal disease in young Mexican children. Moreover, it reveals the importance of future studies concerning the surveillance and molecular epidemiology of gastroenteric viruses and to our knowledge is likely to represent the first global view on the frequency of HuCV infection across Mexico.

## Methods

### Specimen collection

Fecal samples were taken from a collection of specimens obtained through the RNLSP, from different geographical regions of Mexico (North, Central, and South). Stool samples were from children up to 5 years of age with acute gastroenteritis between October 2005 and December 2006, and these were subsequently sent to the Gastrointestinal Viruses Laboratory at the Institute of Epidemiological Diagnosis and Reference (InDRE, its acronym in Spanish) for the intentional search for HuCV and other enteropathogens.

The study included 14/31 states throughout Mexico (Chihuahua, Nuevo León, Tamaulipas, Nayarit, Colima, Michoacán, the Mexico City Federal District, Hidalgo, Puebla, Veracruz, Chiapas, Campeche, Yucatán, and Quintana Roo). All stool samples included in this study were tested previously by the RNLSP against rotavirus by polyacrylamide gel electrophoresis (PAGE) and for other enteric pathogens (data not shown), and were referred for their quality assurance and reference by ELISA (ProSpecT™ Rotavirus, Oxoid Ltd, UK) and RT-PCR for rotavirus at the InDRE Gastrointestinal Viruses Laboratory. According the differential algorithm to acute diarrheal illness (EDA, its acronym in Spanish), the samples were tested for NoV diagnosis by ELISA (IDEIA™ Norovirus, Oxoid Ltd, UK) and further genotyping by RT-PCR.

### RNA extraction and RT-PCR

Viral RNA was extracted from 150 μL of 10-20% stool suspensions in phosphate-buffered saline with the QIAmp Viral RNA Kit (Qiagen GmbH, Hilden, Germany) according to the manufacturer's instructions. RNA was stored at -20°C until its use. To determine the presence of HuCV, one RT-PCR was performed using the Titan One Tube RT-PCR System (Roche Applied Science, Mannheim, Germany) in a 50 μL reaction mixture with a primer set that targeted the RNA polymerase gene reported by Farkas et al. [14]. Afterward, all HuCV-positive samples were further characterized by RT-PCR employing a specific second primer set for detection of NoV GI and NoV GII with primers targeted toward the capsid gene, as has been previously reported [37,38].

### DNA sequencing and data analysis

Amplification products were purified and sequenced from both directions on an ABI PRISM^® ^3100 Genetic Analyzer (Applied Biosystems, Foster City, CA, USA) service supplied by the Laboratory of Genome Pathogens-InDRE. Consensus sequences were obtained by comparing forward and reverse electropherograms utilizing ChromasPro version 1.5 software (Technelysium Pty, Ltd). Database searches were performed employing the Basic Local Alignment Search Tool (BLAST) service supplied by the National Center for Biotechnology Information (NCBI, Bethesda, MD, USA) web server. Both multiple sequence alignments by Clustal W program version 2 [39] and phylogenetic trees with bootstrap analyses from 1,000 replicas and generated by the neighbor-joining method and p-distance as substitution model were performed with MEGA version 5.04 software [40].

The GenBank accession numbers included in the phylogenetic analysis of the *RdRp *of HuCV were the following: AB039781; AF145896; AF182760; AJ011099; AJ249939; AY134748; AY228235; AY032605; AY485642; AY502023; DQ078829; EF126962-EF126964; EF126966; EF363139; EF187497; EF684915; EU096512; FJ537134-FJ537138; GU229659-GU229670; L07418; M87661; U04469; U07611; U22498; X76716; X86557; X86560; X81879; U95643, and U73124, whereas the accession numbers included in the phylogenetic analysis for the *VP1 *gene of NoV strains were the following: AB039778; AB039780; AB039781; AB074893; AB212306; AF145896; AF397156; AY038599; AY485642; AY502023; AY588132; AY682549-AY682550; AY682552; AY845056; AY919139; DQ366347; EF126962-EF126964; EF126966; EF187497; DQ078829; EF684915; EU096512; U46500; AY032605; FJ537134-FJ537138; U07611; U22498; X76716 and X86557. The nucleotide sequences obtained in this study were submitted to GenBank with accession numbers FJ214619-FJ214658, JQ082391-JQ082439 and JQ182643-JQ182705.

## Abbreviations

HuCV: Human caliciviruses; NoV: Norovirus; SaV: Sapovirus; RT-PCR: Reverse transcription polymerase chain reaction; NoV GII: Genogroup II of norovirus; GI.2: Genogroup I genotype 2; GI-GV: Genogroup I to Genogroup V; ELISA: Enzyme linked immunosorbent assay; *VP1*: Capsid gene; *RdRp*: Polymerase gene; PAGE: Polyacrylamide gel electrophoresis; EDA: Acute diarrheal illness; BLAST: Basic Local Alignment Search Tool; NCBI: National Center for Biotechnology Information; InDRE: Instituto de Diagnóstico y Referencia Epidemiológico

## Competing interests

The authors declare that they have no competing interests.

## Authors' contributions

The original concept and design described in the manuscript were performed in equal contributions by FG-S, RMR-A, and HG-L. FG-S and RMR-A performed experiments, sequence alignments, and sequence analyses; both contributed equally and should be considered as first authors. RMR-A and HG-L supervised the overall project, providing essential guidance. FG-S, RMR-A, and HG-L drafted and edited the manuscript. All authors read and approved the final manuscript.
